# The Transcription Factor ZNF395 Is Required for the Maximal Hypoxic Induction of Proinflammatory Cytokines in U87-MG Cells

**DOI:** 10.1155/2015/804264

**Published:** 2015-07-01

**Authors:** Christine Herwartz, Paola Castillo-Juárez, Linda Schröder, Blanca L. Barron, Gertrud Steger

**Affiliations:** ^1^Institute of Virology, University of Cologne, Fürst-Pückler-Strasse 56, 50935 Cologne, Germany; ^2^Escuela Nacional de Ciencias Biológicas, Instituto Politécnico Nacional, 11340 Ciudad de México, DF, Mexico

## Abstract

Hypoxia activates the expression of proangiogenic and survival promoting factors as well as proinflammatory cytokines that support tissue inflammation. Hypoxia and inflammation are associated with tumor progression. The identification of the factors participating in the hypoxia associated inflammation is essential to develop strategies to control tumor hypoxia. The transcription factor ZNF395 was found to be overexpressed in various tumors including glioblastomas particularly in the network of a hypoxic response pointing to a functional role of ZNF395. On the other hand, ZNF395 was suggested to have tumor suppressor activities which may rely on its repression of proinflammatory factors. To address these conflictive observations, we investigated the role of ZNF395 in the expression of proinflammatory cytokines in the astrocytoma cell line U87-MG under hypoxia. We show that ZNF395 is a target gene of the hypoxia inducible factor HIF-1*α*. By gene expression analysis, RT-PCR and ELISA, we demonstrated that the siRNA-mediated suppression of ZNF395 impairs the hypoxic induction of IL-1*β*, IL-6, IL-8, and LIF in U87-MG cells. At ambient oxygen concentrations, ZNF395 had no enhancing effect, indicating that this transcriptional activation by ZNF395 is restricted to hypoxic conditions. Our results suggest that ZNF395 contributes to hypoxia associated inflammation by superactivating proinflammatory cytokines.

## 1. Introduction

Limiting level of oxygen, that is hypoxia, is a characteristic of fast growing solid tumors due to the elevated growth rate. Tumor hypoxia induces adaptive mechanisms to promote survival, angiogenesis, and remodeling of the extracellular matrix. The activation of the family of the hypoxia inducible factors HIF-1*α* and HIF-2*α*, the intracellular sensors for O_2_, plays a crucial role. HIF-1*α* supports cancer progression, metastasis, and chemoradiation resistance mainly through the induction of proangiogenic, survival promoting, and proinflammatory factors that cause chronic inflammation [1]. Chronic inflammation generally promotes malignant progression which led to the postulation of the inflammatory microenvironment as the seventh hallmark of cancer [2]. A hypoxic microenvironment and a clinical significant inflammation are characteristics for glioblastoma multiforme (GBM) which is the most common primary malignant brain tumor. Patients have a poor prognosis with a median overall survival in the range of 15 months [3, 4]. Proinflammatory factors including IL-1*β*, IL-6, and IL-8 were found to be overexpressed in human solid glioblastomas compared to the host normal tissue [5, 6]. The hypoxic treatment of GBM cells resulted in the overexpression of these genes, which correlated with a gain of invasive and migratory capabilities indicating that the proinflammatory cytokines functionally contribute to disease progression [7].

A previous study found the barely characterized transcription factor ZNF395 overexpressed in GBM along with hypoxia-induced genes involved in angiogenesis and inflammation which correlated with a poor prognosis for the patients [8]. ZNF395 was also described as a hypoxia-inducible gene in various glioblastoma, as well as other tumor cell lines [8, 9], and was found to be activated by cyclic and chronic hypoxia [10]. Increased expression of ZNF395 could be associated with a poor prognosis in patients with osteosarcomas, Ewing sarcomas [11, 12], and neuroblastomas [13] and may also play a role in the pathogenesis of clear cell renal cell carcinoma (ccRCC) [14–16]. These observations support a functional role of ZNF395 in the response of tumor cells to hypoxia and in the pathogenesis of these cancers.

On the other hand, tumor suppressing activities were assigned to ZNF395. The downregulation of ZNF395 by MiR-525-3p was shown to enhance migration and invasion of liver cancer cells [17]. Transcripts for ZNF395 and APP (amyloid precursor protein) were identified as targets of the metastasis associated protein TARBP2 in the highly metastatic breast cancer cell line MDA-LM2. Binding of TARBP2, which is a double-stranded RNA binding protein, to transcripts for ZNF395 and APP resulted in their destabilization. Sh-RNA-mediated suppression of ZNF395 in these cells was correlated with metastasis and invasion as well as increased expression of the IL-1*β*, IL-8, and Cox 2 indicating that ZNF395 acts as a transcriptional repressor of these factors [18].

We identified ZNF395 by its ability to repress transcription of some papillomaviruses [19, 20]. The protein was also found to inhibit the Huntington disease (HD) gene promoter [21, 22]. We have shown previously that ZNF395 can act as repressor or activator of transcription depending on the promoter and the cellular environment. The repression of transcription by ZNF395 depended on the recruitment of SIN3A/HDAC1 complex via a direct interaction of ZNF395 with SAP30, a component of this corepressor complex [20]. Beyond that, we deciphered that ZNF395 is required for the maximal interferon *α* (IFN*α*) mediated induction of IFIT1, IFIT2, and IFI16, which was dependent on the activity of the I*κ*B-kinase (IKK) [23].

Here, we address the input of ZNF395 to the expression of proinflammatory cytokines under hypoxia in the well characterized U87-MG cell line established from a GBM patient [24].

## 2. Materials and Methods 

### 2.1. Cell Culture

The human skin cancer cell line RTS3b was described in [25] and kindly provided by I. Leigh, University College, London, UK. RTS3b cells were maintained in E-medium; the monocytic cell line U937 [26] in RPMI1640, U87-MG, HEK293, and C33A cells were cultivated in DMEM. All media were supplemented with 10% FCS and antibiotics. All cells were cultivated in ambient O_2_ concentrations, 5% CO_2_, and 90% humidity. Hypoxia was induced by gassing N_2_ into the incubator which displaced the oxygen to a concentration of 2%. After this was reached, the cells were incubated for 12 hours and total RNA or protein was isolated immediately.

### 2.2. Preparation of Cell Extracts and Western Blotting

The cells were washed twice in ice-cold phosphate-buffered saline and scraped off in LSDB buffer (50 mM Tris-HCl [pH 7.9], 10% glycerol, 0.5 mM EDTA, 1 mM dithiothreitol, and 0.2% NP-40) containing 100 mM KCl and the protease inhibitors PMSF (1 mM), aprotinin, pepstatin, and leupeptin (0.1 mM each) followed by ten 30 seconds of sonication cycles using a Bioruptor (Diagenode). Cell debris was removed by centrifugation for 10 min at 4°C. 60 *μ*g of the total cell extracts was used for Western blotting to detect ZNF395 with a polyclonal antibody generated in rabbits [19] and HIF-1*α* with the rabbit monoclonal antibody EP1215Y (Epitomics, California, USA).

### 2.3. RNA Interference

Small interfering RNAs (siRNA, siGenome SMARTpool) were obtained as a pool of four annealed double-stranded RNA oligonucleotides from Dharmacon, ZNF395 (M-020387), HIF-1*α* (M-004018), and siControl (D-0012061420). Six wells or six cm dishes of U87-MG cells were transfected with 150 pmol siRNA using Lipofectamine RNAiMAX (Invitrogen). The cells were either harvested 48 hours later or were set 36 hours after transfection to 2% O_2_ for another 12 hours. When indicated, the cells were incubated 24 hours after transfection in medium containing 1 mmol DMOG or the equivalent volume of ETOH, the solvent for DMOG, for 24 hours. Total RNA was isolated and the supernatant was collected and stored at −80°C until use.

### 2.4. ELISA

The amount of the cytokines IL-6 and IL-8 present in the supernatant of U87-MG cells was determined with the human IL-6 ELISA Max Standard Set or the IL-8 ELISA Max Standard Set from BioLegend (San Diego, USA) according the manufacturer's instruction.

### 2.5. RT-PCR, Microarray

Total RNA was prepared by the RNeasy Mini Kit from Qiagen or from Macherey-Nagel (Düren, Germany). cDNA synthesis and hybridization to Affymetrix Exon 2.0 ST array was performed by the group of Prof. Nürnberg (CCG, Cologne, Germany). The raw data were processed with the help of the Affymetrix Expression and Transcriptome analysis console. For quantitative real time PCR, 2 *μ*g of RNA was reverse transcribed using random primer and Go-Script Reverse Transcriptase Kit (Promega). Real time PCRs were performed with SYBR Green and a Roche LightCycler 480 (Roche Diagnostics). The expression of the various factors was normalized against the housekeeping gene hypoxanthine guanine phosphoribosyltransferase (HPRT). The values obtained for cells transfected with control siRNA and grown under normoxia were set as 1 and the fold differences were calculated according to the comparative threshold method [27]. The primers that were used for RT-PCR are summarized in Table 1.

### 2.6. Statistical Analysis

**Figure 1:**
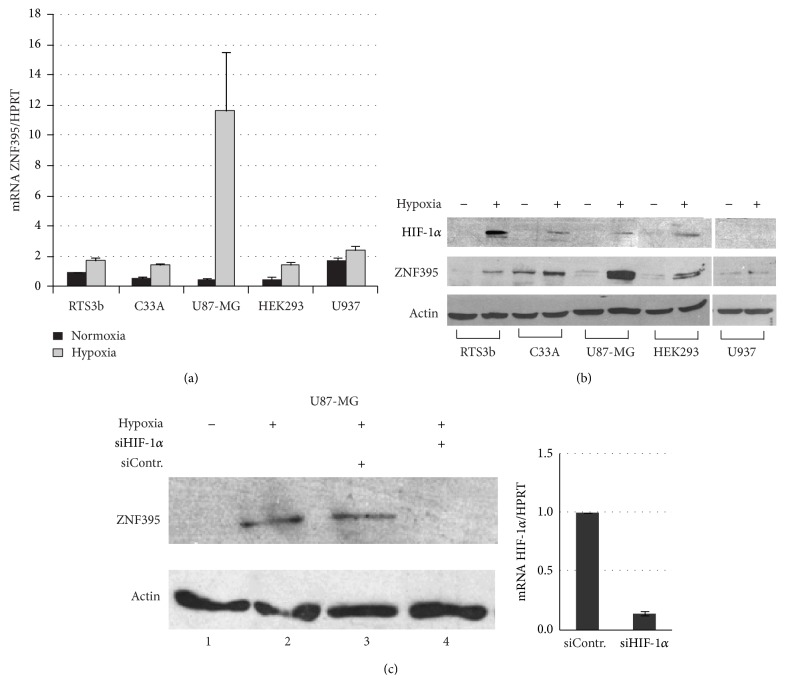
RTS3b, C33A, HEK293, U87-MG, and U937 cells were either grown under ambient O_2_ atmosphere or in the presence of 2% O_2_ for 12 hours before total RNAs or total protein extracts were prepared. (a) Quantitative real time RT-PCR was performed with each RNA and primers specific for ZNF395 and HPRT, which served as house-keeping gene. The ZNF395 values for RTS3b grown at ambient atmosphere were arbitrary set as 1 and the fold activations obtained for all other cells were calculated according to the comparative threshold method as previously described [27]. (b) A Western blot with 60 *μ*g protein extracts from each cell line grown under normoxia or hypoxia was developed with antibodies against HIF-1*α*, ZNF395, or actin, which served as loading control. (c) U87-MG cells were either left untransfected (lanes 1, 2) or transfected with control siRNA (lane 3) or siRNA against HIF-1*α* (lane 4). 36 hours later, the cells were set to hypoxia for 12 hours, as indicated, before protein extracts were prepared and a Western blot with antibodies against ZNF395 and actin was performed. One set of cells was transfected with siControl or siHIF-1*α* and 48 hours later total RNA was isolated, which was used to perform qRT-PCR with primers for HIF-1*α* and HPRT. The fold activation was calculated as in (a).

**Figure 2:**
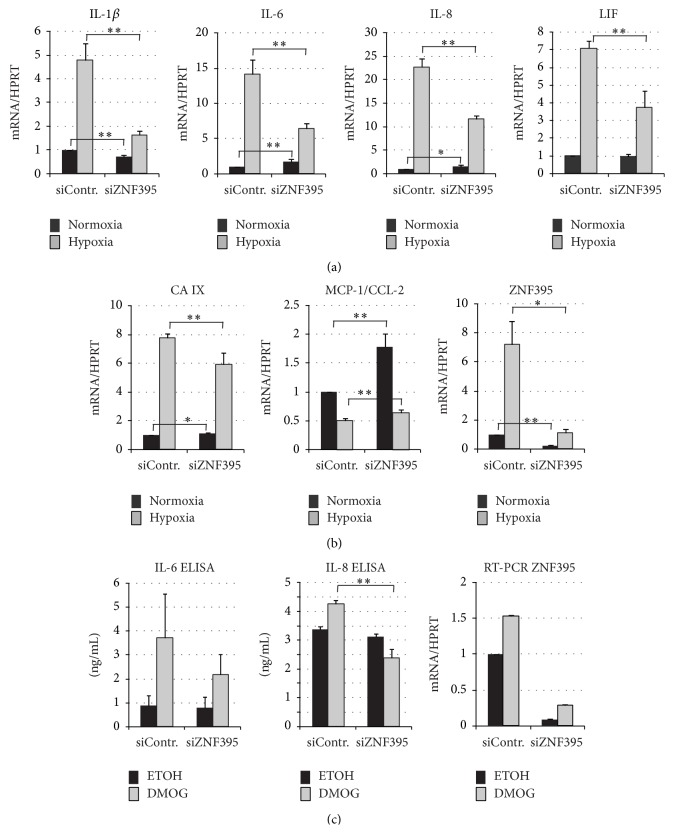
U87-MG cells were transfected with control siRNA or siRNA against ZNF395 and total RNA was isolated 48 hours after transfection (normoxia) or the cells were transferred 36 hours after transfection to 2% O_2_ for additional 12 hours (hypoxia) before total RNA was isolated. cDNA was prepared and quantitative real time-PCRs were performed with primers specific for IL-6, IL-8, IL-1*β*, and LIF (a) and for MCP-1/CCL2, CA IX, and ZNF395 (b) RT-PCR of HPRT serving as internal control. The values obtained by dividing the Ct values for the cytokine and HPRT, respectively, from the siControl cells grown under normoxia were set as 1, and the fold activations were calculated. The standard deviations are given. Each PCR was performed six times in duplicate except that for ZNF395. (c) U87-MG cells were transfected with siControl or siZNF395; 24 hours later, one set of cells was incubated in the presence of DMOG, while the other set of cells obtained the equivalent amount of ETOH (which served as solvent for DMOG) as control. Another 24 hours later, the supernatant was used for ELISA to measure the level of IL-6 and IL-8. Each ELISA was performed two times in triplicate. QRT-PCR with RNA isolated from these cells was used to investigate the effect of DMOG on the expression of ZNF395. ^*∗*^ *p* < 0.05; ^*∗∗*^ *p* < 0.01 by Student's *t*-test.

Enrichment analysis for the gene ontology categories was done by submitting the gene list to the gene-annotation enrichment analysis, functional annotation clustering tool DAVID (http://david.abcc.ncifcrf.gov/). The adjusted *p* value reflects the significance of enrichment. Q-RT-PCRs presented in Figure 1 were performed three times and those in Figures 2(a) and 2(b) were performed six times in duplicate. The significance of the differences between the samples was assessed by Student's *t*-test with paired samples.

## 3. Results

### 3.1. ZNF395 Is Induced by Hypoxia in Various Cancer Cell Lines and Is a Target Gene of HIF-1*α*

Firstly, we compared the magnitude of the induction of ZNF395 by hypoxia in several tumor cell lines, which were RTS3b cells, an immortalized skin cancer cell line, the cervical cancer cell line C33A, the astrocytoma cell line U87-MG, the human epithelial kidney cell line HEK293, and the monocyte cell line U937. The cells were incubated under normal atmosphere, which is 19% O_2,_ or set to 2% O_2_ for 12 hours. QRT-PCR revealed an upregulation of the amounts of ZNF395 transcripts in all cell lines by hypoxia albeit to a different degree. While in the monocytic U937 cells and in the keratinocyte cell line RTS3b the transcript level marginally rose by 1.3- or 1.5-fold in response to hypoxia, respectively, the induction was more than 20-fold in U87-MG cells (Figure 1(a)). A Western blot (WB) with extracts from these cells confirmed the hypoxic induction of the ZNF395 protein in all cell lines. Again, the highest amount of ZNF395 was present in U87-MG cells grown under hypoxia, while in U937 we hardly could detect the protein, independent of the O_2_ concentration (Figure 1(b)). Thus, the protein level of ZNF395 correlated with the amount of mRNA. In the presence of sufficient oxygen, prolyl hydroxylases (PHDs) hydroxylate two proline residues in the HIF*α* subunits. This creates recognition motifs for the von Hippel-Lindau protein (VHL) which is the substrate recognition subunit of an E3 ubiquitin ligase complex that promotes the accelerated degradation of two HIF*α* family members, HIF-1*α* and HIF-2*α*. Oxygen deficiency leads to the inhibition of PHDs with the consequence that the stability of HIF*α* factors increases [28]. Reprobing the WB with an antibody against HIF-1*α* confirmed the increase of the HIF-1*α* protein level upon hypoxia in these cell lines, except for U937 where the amount of HIF-1*α* may be below the detection limit of the antibody (Figure 1(b)).

To address the role of HIF-1*α* in the induction of ZNF395 under hypoxia directly, we transfected U87-MG cells with siRNA against HIF-1*α* and subjected the cells to hypoxia for 12 hours. The WB in Figure 1(c) shows that in the presence of the control siRNA the level of ZNF395 was not affected in hypoxic cells. However, upon transfection of siRNA against HIF-1*α*, ZNF395 could not be detected. Since HIF-1*α* was hardly visible in extracts from U87-MG cells grown under hypoxic conditions (see Figure 1(b)), we confirmed the efficiency of the knock down of HIF-1*α* by qRT-PCR (Figure 1(c)). From these results, we concluded that ZNF395 is a target gene of HIF-1*α*.

### 3.2. Identification of ZNF395 Regulated Genes upon Hypoxia

To systematically investigate the contribution of ZNF395 to the hypoxia regulated gene expression in U87-MG cells, we identified differentially expressed genes with Affymetrix HuGene 2.0 ST gene chips. The expression of 44 genes was activated and 61 genes were repressed by more than 1.5-fold with *p* values below 0.05 due to the siRNA-mediated suppression of ZNF395 in U87-MG cells which were kept under hypoxia for 12 hours. We submitted the list of genes activated by ZNF395 to the functional annotation tool DAVID. The results are shown in Table 2. A significant enrichment of genes involved in the response to wounding was found. The affected genes were NLRC4 (Nod-like-receptor family 4, a component of the inflammasome that activates Caspase 1 and leads to the maturation of IL-1*β* [29]), IL-1*β*, IL-6, SERPINA10 (serpin peptidase inhibitor, clade A, member 10), LIPA (lysosomal acid lipase A), MTPN (myotrophin), C1S (subunit of the C1 complement complex), TIMP3 (tissue inhibitor of metallo proteinases 3), and PLAU (plasminogen activator urokinase). SERPINA10, MTPN, TIMP3, and PLAU belonged to the biological processes tissue regeneration, development growth, and wound healing as well. The latter also included IL-6 and IL-1*β*. The genes repressed by ZNF395 did not show any significant enrichment to biological processes (data not shown).

### 3.3. ZNF395 Is Required for the Maximal Hypoxic Induction of IL-1*β*, IL-6, IL-8, and LIF

One of the most affected genes in our screen was that for IL-1*β* with a 2.13-fold reduction of its expression upon the suppression of ZNF395 (data not shown). Our screen also revealed a reduced expression of IL-8 (1.3-fold, *p* = 0.0015; although, it is below our threshold), the closely related IL-6 (1.49-fold, *p* = 0.015), and the leukemia inhibitory factor (LIF) (1.6-fold, *p* = 0.00278) indicating that ZNF395 activates the expression of IL-1*β*, IL-6, IL-8, and LIF under hypoxic conditions in U87-MG cells. IL-8, IL-1*β*, IL-6, and LIF are hypoxia-induced genes that are associated with hypoxia-mediated inflammation [30–32]. To confirm the contribution of ZNF395 to the control of the expression of these cytokines under limiting oxygen concentrations, we transfected U87-MG cells with siZNF395 or siControl and kept one set of cells under normoxia for another 48 hours, while the second set was incubated for the last 12 hours under 2% O_2_ before isolating RNA. In this experiment, hypoxia activated ZNF395 expression 7.2-fold in the siControl cells, which was reduced to 1.13-fold upon transfection of siZNF395 compared to siControl cells under normoxia (Figure 2(b)). QRT-PCR revealed that hypoxia increased the transcript level of IL-1*β* by 4.9-fold, confirming the hypoxic induction of IL-1*β*, which was shown to require HIF-1*α* [31]. In the siZNF395 cells, the hypoxic induction of IL-1*β* was only 1.9-fold, representing a 2.69-fold decrease due to the lack of ZNF395 (*p* < 0.001). Under normoxic condition, IL-1*β* level dropped to 0.7 after suppression of ZNF395 (*p* < 0.001) (Figure 2(a)).

Similarly, the genes for IL-6 and IL-8 were efficiently induced by hypoxia in the siControl cells, which was 12-fold for IL-6 and 23-fold for IL-8 in accordance with the published data. The lack of ZNF395 impaired this induction by more than 50% in both cases, indicating that ZNF395 is required for full hypoxic induction of IL-6 and IL-8 (both *p* < 0.001). In contrast, under normal conditions, siRNA-mediated suppression of ZNF395 stimulated the expression of IL-6 and IL-8 by 1.4- and 1.7-fold, (*p* < 0.001 and *p* < 0.05), respectively. Thus, ZNF395 may act as a suppressor of these genes under normoxia.

LIF expression was increased 7-fold upon hypoxia in siControl transfected cells. The lack of siZNF395 reduced the hypoxic induction of LIF to 3.8-fold (*p* < 0.001) and had no effect on LIF expression when cells were grown under normoxia, indicating that ZNF395 is required for the full hypoxic induction of LIF as well, but it seems not to be involved under normoxia (Figure 2(a)).

In addition, we analyzed the expression of carbonic anhydrase IX (CA IX), a well known target gene of HIF-1*α*. QRT-PCR demonstrated that in our system CA IX was induced by 7.8-fold upon hypoxia in siControl-cells and 6.0-fold (*p* = 0.004) when ZNF395 was suppressed (Figure 2(b)). Thus, ZNF395 is not a modulator of the transcriptional activity of HIF-1*α* per se, since the expression of CA IX was only marginally reduced upon the lack of ZNF395.

Although hypoxia activates a number of genes to adapt to the metabolic demands under low oxygen, hypoxia leads to global downregulation of transcription. To directly investigate an effect of ZNF395 on gene repression by hypoxia, we focused on the monocyte chemoattractant protein 1 (MCP-1/CCL2). The hypoxia-mediated repression of MCP-1/CCL2 was found to rely on the level of mRNA elongation and to involve the inhibition of the elongation factor P-TEFb [33, 34]. Hypoxia resulted in a 50% reduction of the mRNA-level for MCP-1 in both the siControl and the siZNF395 transfected U87-MG cells. Under normoxia, MCP-l expression increased 1.8-fold upon suppression of ZNF395, indicating that ZNF395 acts as a repressor of MCP-1 under normal growth conditions (Figure 2(b)).

### 3.4. ZNF395 Is Required for the Maximal Induction of IL-6 and IL-8 by the PHD Inhibitor DMOG

The PHD inhibitor dimethyloxalylglycine (DMOG) leads to the stabilization of HIF-1*α* and results in increased amounts of ZNF395 independent of the O_2_ deficiency [9, 22]. To confirm the effect of ZNF395 on the production of IL-6 and IL-8, we treated siZNF395 and siControl transfected U87-MG cells with DMOG for 24 hours. An ELISA with the supernatant of the cells revealed an induction of IL-6 and IL-8 upon incubating the cells with DMOG. These concentrations were reduced by about 40% upon siRNA-mediated suppression of ZNF395 (Figure 2(c)), confirming the requirement of ZNF395 for the full induction of IL-6 and IL-8 under conditions of hypoxia.

## 4. Discussion 

Our data presented here imply that ZNF395 is required for the maximal hypoxic induction of proinflammatory cytokines since the lack of ZNF395 impaired the hypoxia-induced activation of IL-1*β*, IL-6, IL-8, and LIF in U87-MG cells. Our results suggest that the HIF-1*α* induced activation of the expression of ZNF395 is the basis for this effect, at least in part. The notion that ZNF395 is a target gene of HIF-1*α* is in line with a CHIP-on-CHIP experiment that revealed the binding of both HIF-1*α* and HIF-2*α* to two DNA segments which are located several kb upstream of the ORF of ZNF395, respectively [35]. In correlation, the PHD inhibitor DMOG induced the expression of ZNF395 [9, 22, 23, 31, 36]. An upregulation of ZNF395 after hypoxia was described for additional glioblastoma-derived cell lines [8, 9] and most importantly an increased expression of ZNF395 as a part of a hypoxic response was observed in human solid tumors such as GBMs [8] and neuroblastomas [13] implying that also in these in vivo situations ZNF395 enhances the hypoxic induction of these proinflammatory factors and may thus significantly contribute to disease progression. IL-1*β*, IL-6, IL-8, and LIF are well known components of the inflammatory microenvironment, which is important for the initiation and progression of GBM [6]. IL-8 is a potent mediator of angiogenesis and correlates with the histopathological grade of gliomas [5, 37]. Similarly, IL-6 supports invasiveness through the promotion of angiogenesis, cell proliferation, resistance to apoptosis, and radiation of GBM. Targeting IL-6 signaling suppresses glioma stem cell survival and cell growth [38] (reviewed in [5, 39]). Moreover, IL-6 was identified as a critical gene for the activation of the inflammation amplifier in solid tumors [40]. LIF is a multifunctional cytokine with a complex role in cancer. LIF induces the differentiation of several myeloid leukemia cells and inhibits their growth. However, LIF is frequently overexpressed in a variety of human tumors and is associated with a poor prognosis of patients. It promotes tumor progression, metastasis, and chemoresistance in many solid tumors. Hypoxia was shown to induce LIF mRNA expression in human colorectal cancer cells ([41] and references therein). LIF was also correlated with disease outcome of GBM [42]. A contribution of ZNF395 to the development of an inflammatory tumor microenvironment is further supported by the finding that ZNF395 specific mRNA was detected in microvesicles present in the blood from GBM patients [43] and hypoxic glioblastoma cells [44]. Tumor derived microvesicles have been identified as carriers of angiogenic proteins, mRNA, and miRNAs which are thought to suppress the immune response and to accelerate tumor growth by delivering the genetic information to recipient cells in the tumor environment (reviewed in [45]).

Published gene expression analysis also supports the role of ZNF395 as a new factor enhancing hypoxia inflammation for other tumors and diseases. An upregulation of ZNF395 in clear cell renal cell carcinomas (ccRCC) was observed by several groups [14, 15]. Couvé et al. found ZNF395 among the genes whose expression was increased in the ccRCC of patients bearing two gene mutations in the tumor suppressor gene von Hippel-Lindau* VHL*. The upregulation of a cluster of 17 genes including ZNF395 is correlated with the risk of the development of ccRCC implying a functional relevance of ZNF395 in disease progression [16]. A hypoxic induction of ZNF395 was also detected in mature SGBS (Simpson-Golabi-Behmel syndrome) adipocytes. Adipose tissue hypoxia is thought to be involved in the development of obesity-related insulin resistance and chronic inflammation [46].

The here-described results stay in contrast to a previous study suggesting that ZNF395 is a negative regulator of the expression of IL-1*β*, IL-8, and Cox 2. These genes were found to be strongly upregulated upon sh-mediated suppression ZNF395 in breast cancer MDA-LM2 cells under normoxic conditions [18]. Beside the use of other cancer cells that may account for discrepant results, one of the major differences to the study of Goodarzi et al. is that our study addressed the effects of ZNF395 under hypoxia. Under normoxic conditions, the suppression of ZNF395 either had no effect (LIF) or slightly increased (IL-6 and IL-8) the expression of these cytokines in U87-MG cells (Figure 2(a)). Thus, it seems that the effect of ZNF395 is not just dependent on its concentration present in the cell. We speculate that in addition to the induction of its expression, hypoxia-dependent signaling pathways control the transcriptional activity of ZNF395 at the posttranscriptional level. Previously, we demonstrated that the activation of the IFIT1 promoter required the phosphorylation of ZNF395 by IKK. We could further show that ZNF395 undergoes phosphorylation under hypoxia [23]. The inhibition of PHD that occurs also under hypoxia leads to the activation IKK [47, 48] (reviewed in [49]). Thus, it is conceivable that under limiting O_2_ concentrations active IKK will phosphorylate ZNF395 and thus enable ZNF395 to superactivate the expression of the proinflammatory cytokines we have disclosed here.

## 5. Conclusion

Our results demonstrate that the hypoxia inducible transcription factor ZNF395 is a target gene of HIF-1*α*. ZNF395 is required for the maximal hypoxic induction of proinflammatory cytokines Il-1*β*, Il-6, IL-8, and LIF. By enhancing the production of these factors ZNF395 may support hypoxia-induced inflammation and thus contribute to tumor progression.

## Figures and Tables

**Table 1:** Primers used for quantitative real time-PCR.

	Forward primers 5′ to 3′	Reverse primers 5′ to 3′
ZNF395	CGAAAAAAGAAAGAACTCTGTG	CTGTGTCCCCCAGATGGAG
IL-8	ATAAAGACATACTCCAAACCTTTCCAC	AAGCTTTACAATAATTTCTGTGTTGGC
IL-6	GTAGCCGCCCCACACAGA	CATGTCTCCTTTCTCAGGGCTG
IL-1*β*	AAATACCTGTGGCCTTGGGC	TTTGGGATCTACACTCTCCAGCT
LIF	TATCACCATCTGTGCCTTTGCTGC	TCTGCCAGATTGTTCCTATGCCCA
MCP-1	TCGCGAGCTATAGAAGAATCA	TGTTCAAGTCTTCGGAGTTTG
CA IX	TTTGAATGGGCGAGTGATTG	ACAGCAAAAAGGAGGCCAAA
HPRT	TGACACTGGCAAAACAATGCA	GGTCCTTTTCACCAGCAAGCT

**Table 2:** Functional annotation clustering of genes activated (>1.49, *p* > 0.05) by ZNF395 under hypoxia in U87-MG cells that were identified by microarray; annotation clusters with an enrichment score of 2.5 are shown. The adjusted *p* value indicates the significance of enrichment.

Pathway	*p* value	Gene symbol
Response to wounding	3.6 × 10^−5^	NLRC4, IL-6, SERPINA10, LIPA, MTPN, IL-1*β*, C1S, TIMP3, PLAU
Regeneration	6.34 × 10^−5^	LIF, SERPINA10, MTPN, TIMP3, PLAU
Wound healing	3.3 × 10^−4^	IL-6, SERPINA10, MTPN, IL-1*β*, TIMP3, PLAU
Development growth	0.0025	SERPINA10, MTPN, TIMP3, PLAU
